# Effects of Nisin A Combined with Antifungal Drug Against Growth of *Candida* Species

**DOI:** 10.3390/dj13040160

**Published:** 2025-04-08

**Authors:** Yoshie Niitani, Kouji Ohta, Kanako Yano, Yoshino Kaneyasu, Tomoko Maehara, Honami Kitasaki, Hideo Shigeishi, Hiromi Nishi, Rumi Nishimura, Mariko Naito, Fumie Shiba, Miki Kawada-Matsuo, Hitoshi Komatsuzawa, Toshinobu Takemoto

**Affiliations:** 1 Department of Oral Health Management, Program of Oral Health Sciences, Graduate School of Biomedical and Health Sciences, Hiroshima University, 1-2-3 Kasumi, Minami-Ku, Hiroshima 734-8553, Japan; kakiura@hiroshima-u.ac.jp; 2 Department of Public Oral Health, Program of Oral Health Sciences, Graduate School of Biomedical and Health Sciences, Hiroshima University, 1-2-3 Kasumi, Minami-Ku, Hiroshima 734-8553, Japan; otkouji@hiroshima-u.ac.jp (K.O.); d226335@hiroshima-u.ac.jp (K.Y.); yoshi-kane@hiroshima-u.ac.jp (Y.K.); tmaehara@hiroshima-u.ac.jp (T.M.); d245573@hiroshima-u.ac.jp (H.K.); shige@hiroshima-u.ac.jp (H.S.); 3 Department of General Dentistry, Hiroshima University Hospital, 1-2-3 Kasumi, Minami-Ku, Hiroshima 734-8553, Japan; hiyoko@hiroshima-u.ac.jp; 4 Department of Oral Epidemiology, Graduate of School Biomedical and Health Sciences, Hiroshima University, Hiroshima 734-8553, Japan; r-nishimura@hiroshima-u.ac.jp (R.N.); naitom@hiroshima-u.ac.jp (M.N.); 5 Collaborative Research Laboratory of Oral Inflammation Regulation, Graduate School of Biomedical and Health Sciences, Hiroshima University, 1-2-3 Kasumi, Minami-Ku, Hiroshima 734-8553, Japan; fshiba@hiroshima-u.ac.jp; 6 Department of Bacteriology, Graduate School of Biomedical and Health Sciences, Hiroshima University, Hiroshima 734-8551, Japan; mmatsuo@hiroshima-u.ac.jp (M.K.-M.); komatsuz@hiroshima-u.ac.jp (H.K.)

**Keywords:** *Candida*, nisin A, antifungal drugs, combined effect

## Abstract

**Background/Objectives:** Nisin A, an antimicrobial peptide produced by *Lactococcus lactis*, primarily shows antimicrobial activity against Gram-positive bacteria, with efficacy increased when used in combination with an antimicrobial drug. On the other hand, oral candidiasis, caused by *Candida*, occurs in immunocompromised patients and requires antifungal therapy. However, antifungal drug-resistant *Candida* strains are increasing worldwide, leading to serious problems. **Methods:** To examine the effects of nisin A against *Candida* species, we investigated the combined effects of nisin A and antifungal drugs on the growth and viability of *Candida* strains. **Results:** While nisin A alone had no antifungal effect, together with amphotericin (AMPH), it showed synergistic effects towards *C. albicans*, as well as the non-albican strains *C. glabrata*, *C tropicalis*, and *C. parapsilosis* in checkerboard assay results. Furthermore, nisin A with miconazole (MCZ) or micafungin (MCFG) demonstrated a synergistic or additive effect on those strains. Cell viability assay results showed that nisin A enhanced the fungicidal activity of AMPH against both *C. albicans* and *C. glabrata*. Biofilm reduction assays showed that nisin A with AMPH, MCZ, or MCFG inhibited biofilm activity against *C. albicans* as compared with each antifungal drug alone. Finally, nisin A with AMPH, MCZ, or MCFG resulted in a reduced minimum inhibitory concentration of those antifungal drugs against clinically isolated *C. albicans* and *C. glabrata.* **Conclusions:** When used in combination with nisin A, the antifungal drug dosage can be lowered, thus helping to prevent adverse side effects and the emergence of drug-resistant oral *Candida* species.

## 1. Introduction

Nisin is an antimicrobial peptide produced by *Lactococcus* and *Streptococcus* species [1,2]. The originally described variant, known as nisin A, is a class-I bacteriocin low molecular weight pentacyclic antimicrobial peptide produced by *Lactococcus lactis* with 34 amino acid residues [3,4]. Nisin A mainly shows activity against Gram-positive bacteria, including food-borne pathogens such as *Listeria monocytogenes* and *Staphylococcus aureus* [4], and has been approved as safe for human consumption by the World Health Organization and Food and Drug Administration (FDA), and commonly used as a food preservative, especially in dairy products [5,6]. *Candida* is a commensal microorganism often found in the oral cavity of healthy individuals [7]. However, some species, most commonly *Candida albicans*, can cause oral candidiasis, an oral mucosal infection, with the condition generally obtained secondary to immune suppression, which can be local or systemic, with causative factors including age, immunocompromising diseases such as human immunodeficiency virus/acquired immunodeficiency syndrome (HIV/AIDS), and chronic systemic steroid and antibiotic use reported [8,9]. Notably, anti-cancer therapy has been found to frequently cause an immunocompromised condition, leading to oral candidiasis [10], which can occasionally spread into the larynx, pharynx, or esophagus and cause candidemia, a severe blood infection, in affected patients [11]. The mortality rate for candidemia is high, ranging from approximately 30% to 60%. Thus, antifungal drugs are needed for the prompt treatment of individuals with oral candidiasis [12,13]. However, antifungal drug-resistant *Candida* strains have been increasing worldwide, leading to serious problems in clinical settings.

One method proposed to combat the rise in antifungal drug resistance is the use of an antifungal drug and antimicrobial peptide combination that exhibits synergistic or additive effects [14]. An additional possible effect of such synergistic combinations is a decrease in the required dosage of the antifungal drug, which could possibly reduce adverse side effects [15]. Previous results have shown the synergistic effects of nisin when used in combination with an antimicrobial drug on Gram-positive and -negative bacteria [16,17,18,19]. Although growth inhibitory effects of nisin Z (analogous to nisin A: one amino acid substitution with asparagine at position 27 of histidine) on *Candida* species have been reported [20,21], those of nisin A when used in combination with antifungal drugs against *Candida* are not well understood.

Based on a hypothesis stating that nisin A enhances the effect of antifungal drugs against candida, the present study was conducted to examine the effects of nisin A used in separate combinations with amphotericin (AMPH), miconazole (MCZ), and micafungin (MCFG), three antifungal drugs with different mechanisms of action, for treatment against *Candida* species.

## 2. Materials and Methods

### 2.1. Microorganisms and Growth Conditions

*Candida albicans* IFM40009, *C. glabrata* IFM54350, *C. tropicalis* IFM46821, and *C. parapsilosis* IFM5774 were obtained from Chiba University Research Center for Pathogenic Fungi and Microbial Toxicoses, and *C. albicans* IFO1385 was obtained from RIKEN Bioresource Research Center. Seventeen strains of *C. albicans*, including clinical strains #1–17 and nine strains of *C. glabrata*, including clinical strains #1–9, were isolated from patients who visited the Department of General Dentistry for oral care while undergoing cancer treatment after obtaining informed consent for acquisition according to a protocol approved by the Ethical Committee of Hiroshima University (E-3139). Cultures positive for the presence of *Candida* species were selected based on colorimetric observation of colonies grown in CHROMagar (Kanto Chemical, Tokyo, Japan) chromogenically converted by species-specific enzymes contained in *Candida* species.

Each *Candida* species was grown in Sabouraud dextrose agar (Becton, Dickinson and Company, Cockeysville, MD, USA) at 37 °C for 48 h; then, the resulting colonies were added to a saline solution, with the concentration adjusted to 0.5 on the McFarland scale (approximately 1–5/10^6^ cells/mL). *Candida* undergoes a transition from a yeast to hyphal form in a growth condition. The morphology of *C. albicans* and *C. tropicalis* transform from yeast to pseudohyphae and hyphae, and *C. parapsilosis* grows to a pseudohyphal form, whereas *C. glabrata* growth occurs in a yeast morphology because of its inability to *form hyphae* [22]. The hyphae form has been found to be promoted when grown in RPMI1640 medium [23], and we have confirmed hyphae forms of *C. albicans*, *C. tropicalis*, and *C. parapsilosis* when grown in RPMI1640 medium (Nacalai Tesque Inc., Kyoto, Japan) with morpholine propanesulfonic acid (MOPS) using microscope.

### 2.2. Peptide and Antifungal Drugs

Nisin A (2.5% purity, 1000 U/mg) was purchased from Sigma-Aldrich (St. Louis, MO, USA). AMPH, MCZ, and MCFG, antifungal drugs used for this study, were purchased from Cayman Chemical Company (Ann Arbor, MI, USA).

### 2.3. Checkerboard Assay

First, the minimum inhibitory concentration (MIC) of each antifungal drug and nisin A were determined using a growth inhibitory assay, with the details described in another section. Nisin A solution with a two-fold dilution from 2048 μg/mL did not inhibit the growth of the *Candida* strains. A checkerboard titration method was thus employed with 96-well titer plates (Greiner Bio-One GmbH, Frickenhausen, Germany) using a previously reported method [24,25,26]. Each antifungal drug and nisin A were diluted in RPMI 1640 medium (Nacalai-tesque Inc., Kyoto, Japan), then adjusted to pH 7 using 0.165 M of morpholinepropanesulfonic acid (MOPS). The MIC of nisin A could not be determined; thus, nisin A solutions ranging from 32 to 2048 μg/mL with a two-fold serial dilution were used for this assay, while the MICs of AMPH, MCZ, and MCFG were determined and shown to range from 0.015 to 2 μg/mL, 0.062 to 4 μg/mL, and 0.015 to 4 μg/mL, respectively, depending on the *Candida* species and clinical isolates tested. Aliquots of each drug and nisin A solution (each 100 μL) were dispensed into individual wells of 96-well microplates. Inocula for each *Candida* species were prepared using Sabouraud dextrose agar (Becton, Dickinson and Company, Cockeysville, MD, USA) at 37 °C for 48 h, then the colonies were added to a saline solution, with the concentration adjusted to 0.5 on the McFarland scale (1–5 10^6^ cell/mL). Next, each *Candida* solution was diluted in RPMI 1640 medium and adjusted to pH 7 using MOPS, then 100 uL was added to the 100 uL drug and nisin A solutions for a final concentration of 0.5–2.5 × 10^3^ cells/mL). After 24 h of incubation at 37 °C, the MIC was defined as the lowest concentration of the test agent that inhibited growth by more than 90% as compared with that of the agent-free control, determined based on optical density findings obtained with the use of an iMark microplate reader (Bio-Rad, Hercules, CA, USA). All assays were performed in triplicate.

Originally, the fractional inhibitory concentration (FIC) was calculated as follows: FIC = (MIC of nisin A in combination/MIC of nisin A alone) + (MIC of antifungal drug in combination/MIC of antifungal drug alone) [24,25]. However, nisin A at 2048 μg/mL did not inhibit the growth of the *Candida* strains, and the MIC could not be determined within that concentration. Therefore, the FIC calculation was modified, as shown: modified FIC = (MIC of nisin A in combination/2048 μg/mL) + (MIC of antifungal drug in combination/MIC of antifungal drug alone). The minimum results obtained with those values were defined as modified FIC between antifungal drug and nisin A. Based on previous reports [24,25], synergy was defined as a modified FIC index of ≤0.5, additive effect was defined as a modified FIC index > 0.5 to ≤1, indifference was defined as a modified FIC index > 1 to ≤2, and antagonism was defined as a modified FIC index >2.

### 2.4. Cell Viability Assays

A previously reported method for determining colony-forming units was used [27,28,29] with minor modifications (Supplementary Figure S1). Overnight cultures of *Candida* were harvested and placed into Sabouraud dextrose broth medium (Becton, Dickinson and Company, Cockeysville, MD, USA). After the OD_660_ reached 1.0 (approximately 10^8^ cell/mL), the candida cells were collected and washed with PBS and suspended in 10 mM of sodium phosphate buffer (NaPi) (pH 6.8). The *Candida* suspensions were diluted with NaPi to 10^7^ cells/mL^−1^, then 10 μL of each suspension was inoculated into 200 μL of NaPi along with the appropriate dilution of nisin A and antifungal drug, then incubated for two hours at 37 °C. Thereafter, the reaction mixture was diluted with NaPi and plated in Sabouraud dextrose agar (Becton, Dickinson and Company) and incubated at 37 °C for 48 h. The total number of *Candida* colonies in each plate was counted, and then the percentage of surviving *Candida* as compared to total colony numbers in the control plates (only *Candida* with NaPi, antifungal drug and nisin A not present) was determined.

### 2.5. Biofilm Reduction Assay

A biofilm reduction assay was performed using a metabolic 2,3-bisphosphoglycerate (2-methoxy-4-nitro-5-sulfophenyl)-2*H*-tetrazolium-5-carboxanilide (XTT) combination with a previously reported method, modified as noted following [30]. Overnight cultures of *Candida* were harvested and placed into a Sabouraud dextrose broth medium. After the OD_660_ had reached 1.0 (approximately 10^8^ cell/mL), candida cells were collected, and the cell concentration in the suspension was adjusted to approximately 5 × 10^6^ cell/mL in Sabouraud dextrose broth medium with 8% glucose. Next, 100 μL suspensions were incubated in a flat-bottomed 96-well microtiter plate (Greiner Bio-One) at 37 °C for 24 h. Following biofilm formation, the medium was discarded, and nonadherent cells were removed by thoroughly washing the biofilms three times in sterile PBS. Then, 100 μL was added to a solution of AMPH, MCZ, or MCFG, or each of those antifungal drugs separately combined with 1000 μg/mL of nisin A in RPMI 1640 medium and adjusted to pH 7 with MOPS, after which the biofilms were incubated in the presence of the antifungal agent for 24 h, then washed gently three times in sterile PBS. A 100 μL solution containing XTT (Cayman Chemical Company) at 5 mg/mL and menadione (Sigma-Aldrich) at 1 μM was then added to each well, and the plates were incubated in the dark for two hours at 37 °C, after which colored supernatant from each well was transferred to a fresh microtiter plate and absorbance was measured at 470 nm using a microplate reader. All assays were performed in triplicate.

### 2.6. Growth Inhibition Assay

The procedures for the dilution of broth used for antifungal susceptibility testing of yeasts and filamentous fungi were standardized according to the protocol of the CLSI (M27-A3) [21], with some modifications. Preparations of the antifungal drug alone and in combination with nisin A at 100 μg/mL were diluted in RPMI 1640 medium (Nacalai-Tesque, Inc.) and adjusted to pH 7 with MOPS at 0.165 M. Aliquots of the drug and nisin A solutions (100 μL) were dispensed into each well of 96-well titer plates (Greiner Bio-One). Inocula for each *Candida* species were prepared using Sabouraud dextrose agar (Becton, Dickinson and Company) at 37 °C for 48 h, then the colonies were added to saline solution, with the concentration adjusted to 0.5 on the McFarland scale (1–5 10^6^ cell/mL). Next, each *Candida* solution was diluted in RPMI 1640 medium and adjusted to pH 7 with MOPS, then 100 μL was added to 100 μL of a two-fold dilution solution of AMPH, MCZ, or MCFG, or each of those antifungal drugs combined with 1000 μg/mL of Nisin A at a final concentration of 0.5–2.5 × 10^3^ cells/mL. After 24 h of incubation at 37 °C, the MIC was defined as the lowest concentration of the test agent that inhibited growth by more than 90% as compared to that of the agent-free control, determined based on optical density using an iMark microplate reader (Bio-Rad, Hercules, CA, USA). All assays were performed in triplicate.

### 2.7. Statistical Analysis

The JMP software package, version 16 (SAS Institute, Inc., Cary, NC, USA), was used for statistical analysis. Results were analyzed using a paired Student’s *t*-test and one-way analysis of variance (ANOVA), followed by a Tukey–Kramer post hoc multiple comparisons test. Values are presented as the mean ± SD of three independent experiments.

## 3. Results

The effects of the antifungal drugs, along with nisin A, on the growth of all tested *Candida* species were examined using a checkerboard titration method. Nisin A alone at 2048 μg/mL did not affect the growth of any of the *Candida* species, while that in combination with each of the antifungal drugs showed either additive or synergistic effects (Table 1 and Table 2). Nisin A with AMPH and also with MCZ showed synergistic effects on three *C. albicans* strains tested (Table 1), while that with MCFG had synergistic or additive effects on all of the *C. albicans* strains. Furthermore, nisin A combined with AMPH also had synergistic effects on *C. glabrata*, *C. tropicalis*, and *C. parapsilosis*, and MCZ or MCFG showed synergistic or additive effects on those non-albican strains.

**Figure 1:**
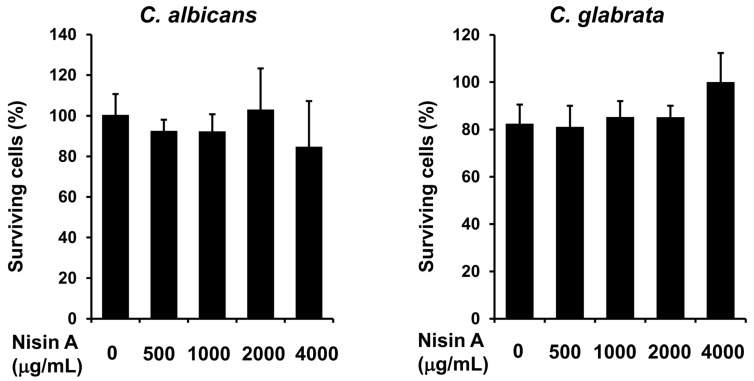
Antifungal activity of nisin A. *C. albicans* IFO1385 and *C. glabrata* IFM54350 were harvested, then washed and suspended in NaPi. Each *Candida* suspension was inoculated into NaPi with the indicated dilution of recombinant nisin A and incubation was performed for two hours at 37 °C. The examined mixtures were then separately plated in agar and incubated at 37 °C for 48 h. The total number of *Candida* colonies in each plate was counted, then the percentage of surviving *Candida* as compared to total colony number in the control plates (*Candida* with NaPi at 0 μg/mL) was determined. Values are presented as the mean ± SD of three independent experiments.

**Figure 2:**
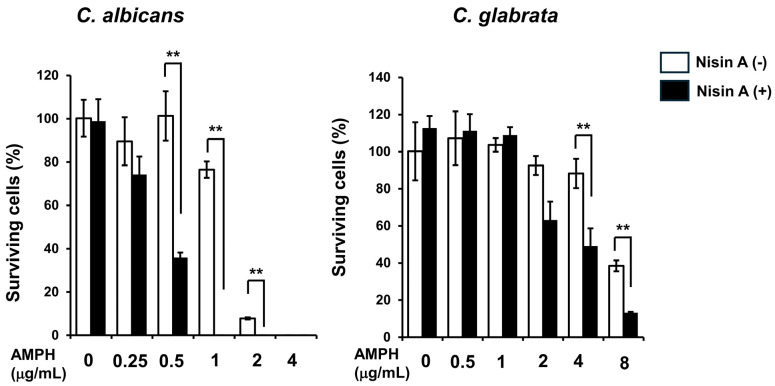
Antifungal activity of combination of nisin A and AMPH. *C. albicans* IFO1385 and *C. glabrata* IFM54350 were harvested, then washed and suspended in NaPi. Each *Candida* suspension was inoculated into NaPi with the indicated dilution of AMPH together with 1000 μg/mL of nisin A, and incubation was performed for two hours at 37 °C. The mixtures were then separately plated in agar and incubated at 37 °C for 48 h. The total number of *Candida* colonies in each plate was counted, then the percentage of surviving *Candida* as compared to total colony number in the control plates (*Candida* with NaPi at 0 μg/mL) was determined. Values are presented as the mean ± SD of three independent experiments. A significant difference between AMPH alone, and the combination of AMPH and nisin A was noted (paired *t*-test. ** *p* < 0.01).

**Figure 3:**
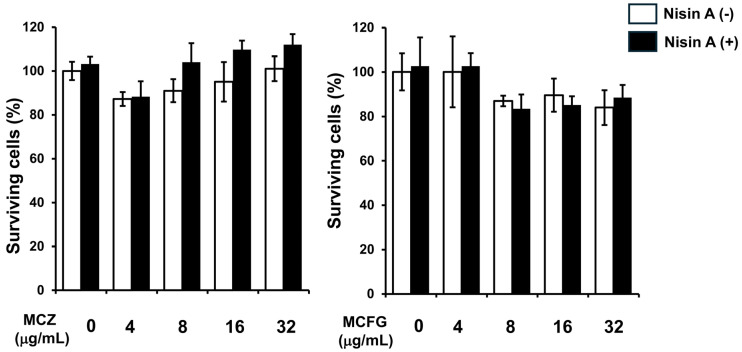
Antifungal activity of combination of nisin A and MCZ or MCFG. *C. albicans* IFO1385 were harvested, then washed and suspended in NaPi. Each *Candida* suspension was inoculated into NaPi with the indicated dilution of MCZ or MCFG together with 1000 μg/mL of nisin A, and incubation was performed for two hours at 37 °C. The mixtures were then separately plated in agar and incubated at 37 °C for 48 h. The total number of *Candida* colonies in each plate was counted, then the percentage of surviving *Candida* as compared to total colony number in the control plates (*Candida* with NaPi at 0 μg/mL) was determined. Values are presented as the mean ± SD of three independent experiments.

Next, the fungicidal effects of nisin A in combination with each of the antifungal drugs on *C. albicans* and *C. glabrata*, which are frequently isolated from oral candidiasis cases and known to cause clinical problems [31], were examined using cell viability assays. Nisin A alone did not show fungicidal activity against either strain (Figure 1). In contrast, nisin A at 1000 μg/mL added to AMPH enhanced the fungicidal activity against both *C. albicans* and *C. glabrata* as compared with the examined antifungal drugs alone (Figure 2). On the other hand, nisin A at 1000 μg/mL added to MCZ or MCFG did not have an effect on the fungicidal activity against *C. albicans* (Figure 3), nor on that against *C. glabrata* (Supplementary Figure S2).

**Figure 4:**
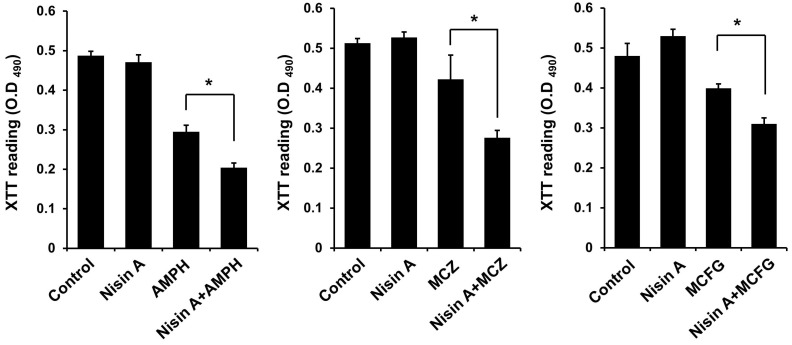
Anti-biofilm activity with combination of nisin A and AMPH, MCZ or MCFG. Biofilm formed by *C. albicans* IFO1385 was harvested, and added to an RPMI 1640 medium solution containing AMPH (0.5 μg/mL), MCZ (0.5 μg/mL), or MCFG (0.062 μg/mL), or each of those antifungal drugs combined with 1000 μg/mL of nisin A, then and incubated at 37 °C for 24 h. OD_490_ values were determined using a biofilm reduction assay. A significant difference between each antifungal drug alone, and the combination of that antifungal drug and nisin A was noted (Tukey–Kramer multiple comparisons test, * *p* < 0.05).

One of the most important virulence factors related to fungus infections involves the formation of biofilm [32]. Especially, *C. albicans* is an intensely biofilm-forming fungus in Candida species [33]. Therefore, we examine the inhibitory effect of nisin A in combination with each of the antifungal drugs on the biofilm activity of *C. albicans*. Although nisin A alone did not show ant-biofilm activity, when it was added to each of the antifungal drugs, biofilm activity was inhibited to a greater degree than with each alone (Figure 4).

**Figure 5:**
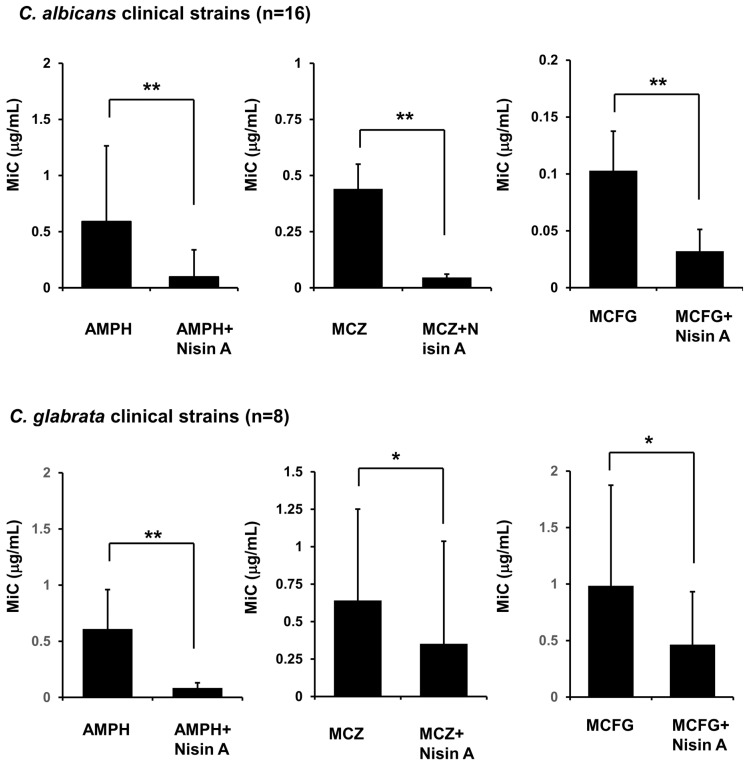
Combined effect of nisin A and antifungal drugs on growth of clinically isolated *Candida* strains. Clinically isolated *C. albicans* (n = 16) and *C. glabrata* (n = 8) strains were harvested. Each strain was separately added to a two-fold dilution of AMPH, MCZ, or MCFG, or each those antifungal drugs and 1000 μg/mL of nisin A at a final concentration of 0.5–2.5 × 10^3^ cells/mL in RPMI 1640 medium buffered to pH 7 with 0.165 M MOPS. After 24 h of incubation at 37 °C, MIC was defined as the lowest concentration of the test agent that inhibited more than 90% growth as compared with the agent-free control. A significant difference between AMPH alone, and the combination of AMPH and nisin A was noted (paired *t*-test. ** *p* < 0.01, * *p* < 0.05).

Finally, the inhibitory effects of nisin A used in combination with each of the antifungal drugs on the growth of *C. albicans* (n = 16) and *C. glabrata* (n = 8) strains isolated from oral cavities of cancer patients were examined using a susceptibility test. Nisin A at 1000 μg/mL alone did not have an effect against any of the clinically isolated *Candida* strains (Supplementary Tables S1 and S2). However, its addition to AMPH, MCZ, or MCFG resulted in a significantly reduced MIC of each of those antifungal drugs against the *C. albicans* and *C. glabrata* strains (Figure 5), and those MIC of antifungal drug or combination of nisin A and antifungal drug against each clinically isolated strains were shown in Supplementary Tables S1 and S2. It was concluded that nisin A enhances the inhibitory effect of antifungal drugs on the growth of *Candida* species.

## 4. Discussion

The activity of nisin A is primarily against Gram-positive bacteria, such as *Bacillus cereus*, *Listeria monocytogenes*, *Enterococci*, *Staphylococci*, and *Streptococci*, while that against Gram-negative bacteria is low [34]. A few studies have suggested that nisin may possess antifungal activity towards *C. albicans*. Of those, Le Lay et al. reported the inhibitory effect of nisin Z on *C. albicans* growth [20], while other findings presented indicate that nisin Z may inhibit *C. albicans* adhesion and the transition of candida to human gingival cells [35]. Furthermore, Gao et al. noted that nisin showed antifungal activity against clinical isolates of azole-resistant *C. tropicalis* strains [21]. In the present study, MIC could not be determined by 2048 μg/mL of nisin A, and the evaluation method was different from that noted in previous reports. On the other hand, previous reports have noted synergistic effects of nisin when used in combination with various antimicrobial drugs against Gram-positive as well as Gram-negative bacteria. For example, nisin in combination with ceftriaxone or cefotaxime showed effects against *Salmonella* [36]. In other studies, the combination of nisin with polymyxin B (PMB) showed synergistic effects against Gram-negative bacteria, including *Pseudomonas aeruginosa*, *Escherichia coli*, *Klebsiella pneumoniae*, and *Pseudomonas putida* [18], while nisin with oxacillin showed such an effect against methicillin-resistant *Staphylococcus aureus* (MRSA) [16]. The present study found synergistic or additive effects of nisin A when administered in combination with antifungal drugs against *Candida* species. These results indicate that nisin A can enhance the growth inhibitory effect of antifungal drugs, even though when used alone, it has a quite low or no antifungal ability against *Candida*.

Another study examined the antibacterial mechanism of nisin against Gram-positive bacteria and found it to bind to lipid II, an essential precursor of the bacterial cell wall, in Gram-positive bacteria, which then interfered with peptidoglycan formation during cell wall synthesis [37], indicating that nisin displays a high level of antibacterial activity against Gram-positive but not Gram-negative bacteria. On the other hand, Lay et al. showed that nisin Z inhibits transformation from the blastospore to hyphal form, leading to ultrastructural disturbances of *C. albicans* [20]. The present results indicated a synergistic or additive effect of nisin A when used in combination with AMPH, MCZ, or MCFG against *Candida* species. It has been shown that AMPH selectively binds to ergosterol in *Candida* cell walls and directly induces pore formation in the cell membrane, which results in disruption of membrane stability [38]. The azole MCZ inhibits the candida ergosterol biosynthesis pathway, thus preventing cell membrane synthesis [39], while MCFG inhibits 1,3-β-D-glucan, an essential cell wall synthesis component [40]. Each of these antifungal drugs has different antifungal functions against the cell membrane or cell walls of *Candida*. Although nisin A up to 2048 μg/mL did not demonstrate growth inhibitory effects against *Candida* species, it may directly induce a low level of damage to a cell component structure, such as the cell wall or membrane, during growth, resulting in enhancement of the effect to inhibit such growth by each drug.

The fungicidal effects of antifungal peptides were examined using cell viability assays. Although nisin A alone did not have effects on the viability of *Candida* cells, when combined with AMPH, fungicidal activity against both *C. albicans* and *C. glabrata* was enhanced as compared with each of those antifungal drugs alone. However, the addition of nisin A to MCZ or MCFG did not affect their fungicidal activities against those strains. The direct fungicidal activity of antifungal drugs and peptides in buffer solution over a two-hour period was examined using the same method, with the antifungal mechanism of AMPH found to directly promote permeability of *Candida* cell membranes, leading to cell death [38]. Therefore, nisin A may also have direct effects on the structure of *Candida* cells, resulting in increased cell membrane destruction when administered with AMPH. On the other hand, MCZ and MCFG have each been shown to inhibit the biosynthesis of *Candida* cell membranes and cell walls [39,40]. The cell viability assay used in the present study evaluates the effects of antimicrobials under non-growth conditions. Thus, some antibiotics may not be effectively assessed as they target growth-related cell behaviors. It is important to note that the fungicidal methods used in the present examinations might not have accurately reflected the antifungal effects of MCZ or MCFG when used together with nisin A.

A major virulence attribute of *C. albicans* is its ability to form biofilm, as it has been reported to be the highest biofilm producer among Candida species [33,41]. The anti-biofilm activities of combinations of nisin A and antifungal drugs against this ability of *C. albicans* were examined in the present study. While nisin A alone did not show anti-biofilm activities, when added to each of the examined antifungal drugs, biofilm activity was inhibited to a greater degree than that of each drug alone. It has also been reported that the biofilm-forming activity of *C. albicans is* associated with hyphae formation growth [42]. The organisms attach to epithelial surfaces and then undergo a transition from yeast to hyphal form *and promote biofilm formation* [43]. It has been noted that antifungal drugs such as AMPH block the hyphae transformation of *C. albicans*, resulting in decreased growth [43], while nisin Z also inhibits the transition from blastospore to hyphae formation [20]. The RPMI1640 medium used in the present study promotes the hyphae form [23]. Our results indicate that nisin A may enhance the inhibitory effects of various antifungal drugs on the morphological transformation of *C. albicans*, as well as its growth and viability, resulting in reduced biofilm activity.

The frequency of candidiasis caused by drug-resistant *Candida* has dramatically risen over the recent two decades, with the increase in clinically isolated *Candida* species considered to be a serious problem [44]. The overuse of existing antifungal drugs has been shown to be related to the development of drug-resistant *Candida* organisms [45]. While the present findings showed that nisin A alone did not have effects on clinically isolated *Candida* strains, it did in combination with AMPH, MCZ, or MCFG, significantly reducing the MIC of those antifungal drugs against both *C. albicans* and *C. glabrata*. Long-term use of antifungal drugs can easily lead to fungal resistance and a prevalence of *Candida* organisms with drug resistance [46] and can increase the risk of various organ injuries as a side effect [47]. Therefore, the combined use of nisin with an antifungal agent may be an important strategy to combat drug-resistant *Candida*, while another benefit could be a reduction in the dosage required for such antifungal drugs, thus possibly decreasing adverse side effects.

The present study has some limitations. Commercially obtained nisin A was used in the present experiments, though previous studies have noted that commercial preparations generally contain 2.5% pure nisin [48,49,50], with the balance of solids consisting of dairy, protein, and salt components [51]. Although currently unavailable from a commercial source, extraction of nisin with the use of a purification method such as chromatography may be necessary to determine the most effective concentration in a future investigation more accurately. In this study, the MIC of nisin A was >2048 μg/mL, though it could not be precisely determined. Thus, the maximum concentration was used as the standard for the calculation of the FIC index, and a modified FIC index was determined. While scores obtained with the use of a modified FIC index may be different from those with the use of a traditional FIC index, the synergistic and additional effects of nisin A together with an antifungal drug can be effectively evaluated using modified FIC index values.

## 5. Conclusions

Nisin A was found to enhance the antifungal effects of AMPH, MCZ, and MCFG. The present results showed that combinations of nisin A with antifungal drugs can lower the required dosage of the drug, thus helping to prevent adverse side effects and the emergence of drug-resistant oral *Candida* species.

## Figures and Tables

**Table 1:** Antifungal effects of nisin A in combination with AMPH, MCZ, or MCFG against *C. albicans*.

*Candida* Species	Antifungal Drug	MIC (g/mL)			* Modified FIC	^#^ Effect
		Antifungal Alone	Antifungal (with Nisin A)	Nisin A (with Antifungal)		
*C. albicans* IFO1385	AMPH	0.5	0.062	64	0.155	Synergy
	MCZ	0.5	0.125	32	0.265	Synergy
	MCFG	0.062	0.031	32	0.515	Additive
*C. albicans* IFM40009	AMPH	0.5	0.031	64	0.093	Synergy
	MCZ	1	0.25	32	0.265	Synergy
	MCFG	0.25	0.062	128	0.310	Synergy
*C. albicans*	AMPH	2	0.5	64	0.281	Synergy
Ca Clinical strain #1	MCZ	0.25	0.062	256	0.373	Synergy
	MCFG	0.125	0.062	32	0.511	Additive

Nisin A alone at up to 2048 μg/mL did not inhibit the growth of any of the examined *Candida* strains. * Modified FIC = (MIC of nisin A in combination/2048 μg/mL) + (MIC of antifungal drug in combination/MIC of antifungal drug alone). ^#^ Combined effect: Synergy was defined as modified FIC index of ≤0.5, additive effect as modified FIC index of >0.5 to ≤1, indifference as modified FIC index of >1 to ≤ 2, and antagonism as modified FIC index of >2.

**Table 2:** Antifungal effects of nisin A in combination with AMPH, MCZ, or MCFG against *non-albicans*.

*Candida* Species	Antifungal Drug	MIC (g/mL)			* Modified FIC	^#^ Effect
		Antifungal Alone	Antifungal (with Nisin A)	Nisin A (with Antifungal)		
*C. glabrata* IFM54350	AMPH	1	0.25	256	0.265	Synergy
	MCZ	1	0.5	32	0.515	Additive
	MCFG	0.125	0.062	256	0.621	Additive
*C. glabrata*	AMPH	1	0.25	32	0.265	Synergy
Cg Clinical strain #1	MCZ	1	0.125	256	0.250	Synergy
	MCFG	0.062	0.031	512	0.750	Additive
*C. tropicalis* IFM46821	AMPH	0.25	0.062	32	0.263	Synergy
	MCZ	2	1	128	0.562	Additive
	MCFG	1	0.5	32	0.515	Additive
*C. parapsilosis* IFM5774	AMPH	0.25	0.062	32	0.263	Synergy
	MCZ	2	1	512	0.750	Additive
	MCFG	1	0.5	32	0.515	Additive

Nisin A alone at up to 2048 μg/mL did not inhibit the growth of any of the examined *Candida* strains. * Modified FIC = (MIC of nisin A in combination/2048 μg/mL) + (MIC of antifungal drug in combination/MIC of antifungal drug alone). ^#^ Combined effect: Synergy was defined as modified FIC index of ≤0.5, additive effect as modified FIC index of >0.5 to ≤1, indifference as modified FIC index of >1 to ≤ 2, and antagonism as modified FIC index of >2.

## Data Availability

All data generated or analyzed in this study are included in this manuscript.

## Supplementary Materials

The following supporting information can be downloaded at https://www.mdpi.com/article/10.3390/dj13040160/s1, Figure S1: Cell Viability Assay; Figure S2: Antifungal activity of combination of nisin A and MCZ or MCFG against *C. glabrata*; Table S1: Minimum inhibitory concentration (MIC) of antifungal drug or combination of nisin A and antifungal drug against clinically isolated *C. albicans* strains; Table S2: Minimum inhibitory concentration (MIC) of antifungal drug or combination of nisin A and antifungal drug against clinically isolated *C. glabrata* strains.
