# Supplementary material for: Effects of Nisin A Combined with Antifungal Drug Against Growth of Candida Species
Source: Dent J (Basel). 2025 Apr 8;13(4):160. doi: 10.3390/dj13040160 (PMC12025931; doi:10.3390/dj13040160)
Supplement: Supplementary file 1 [file dentistry-13-00160-s001.zip › Supplemental Table S1.pdf]

Supplemental Table 1 Minimum inhibitory concentration (MIC) of antifungal drug or combination of nisin A and antifungal drug against clinically isolated *C. albicans* strains.

Strain	MIC (μg/mL)					
	AMPH	AMPH+nisin A	MCZ	MCZ+nisin A	MCFG	MCFG+nisin A
C. albicans #2	0.5	0.031	1	0.062	0.125	0.031
C. albicans #3	0.25	0.031	1	0.031	0.125	0.031
C. albicans #4	0.25	0.031	0.062	0.062	0.062	0.003
C. albicans #5	0.25	0.031	0.125	0.031	0.125	0.062
C. albicans #6	0.5	0.062	0.125	0.031	0.125	0.031
C. albicans #7	0.5	0.062	1	0.031	0.062	0.031
C. albicans #8	0.5	0.062	0.5	0.031	0.062	0.031
C. albicans #9	0.5	0.062	0.031	0.031	0.031	0.004
C. albicans #10	0.5	0.062	0.125	0.031	0.125	0.031
C. albicans #11	0.5	0.062	2	1	0.125	0.062
C. albicans #12	0.5	0.062	1	0.062	0.125	0.062
C. albicans #13	0.5	0.062	0.063	0.031	0.062	0.031
C. albicans #14	0.5	0.031	0.25	0.031	0.125	0.062
C. albicans #15	0.25	0.031	0.125	0.031	0.125	0.015
C. albicans #16	0.5	0.062	2	0.031	0.125	0.015
C. albicans #17	0.5	0.031	0.031	0.031	0.125	0.015

Nisin A at 1000 μg/mL alone did not have an effect against any of the clinically isolated *C. albicans* strains
