# Supplementary material for: Effects of Nisin A Combined with Antifungal Drug Against Growth of Candida Species
Source: Dent J (Basel). 2025 Apr 8;13(4):160. doi: 10.3390/dj13040160 (PMC12025931; doi:10.3390/dj13040160)
Supplement: Supplementary file 1 [file dentistry-13-00160-s001.zip › Supplemental Table S2.pdf]

Supplemental Table 2 Minimum inhibitory concentration (MIC) of antifungal drug or combination of nisin A and antifungal drug against clinically isolated *C. glabrata* strains.

Strain	MIC (μg/mL)					
	AMPH	AMPH+nisin A	MCZ	MCZ+nisin A	MCFG	MCFG+nisin A
C. glabrata #2	0.5	0.062	2	2	1	0.5
C. glabrata #3	1	0.125	0.5	0.062	0.5	0.062
C. glabrata #4	0.125	0.031	0.25	0.062	2	1
C. glabrata #5	0.25	0.021	0.5	0.062	2	1
C. glabrata #6	1	0.125	0.125	0.031	0.125	0.062
C. glabrata #7	1	0.125	0.5	0.062	0.125	0.062
C. glabrata #8	0.5	0.062	1	0.5	2	1
C. glabrata #9	0.5	0.125	0.25	0.031	0.125	0.031

Nisin A at 1000 μg/mL alone did not have an effect against any of the clinically isolated *C. glabrata* strains
