# Supplementary figures and images for: Effects of Nisin A Combined with Antifungal Drug Against Growth of Candida Species
Source: Dent J (Basel). 2025 Apr 8;13(4):160. doi: 10.3390/dj13040160 (PMC12025931; doi:10.3390/dj13040160)

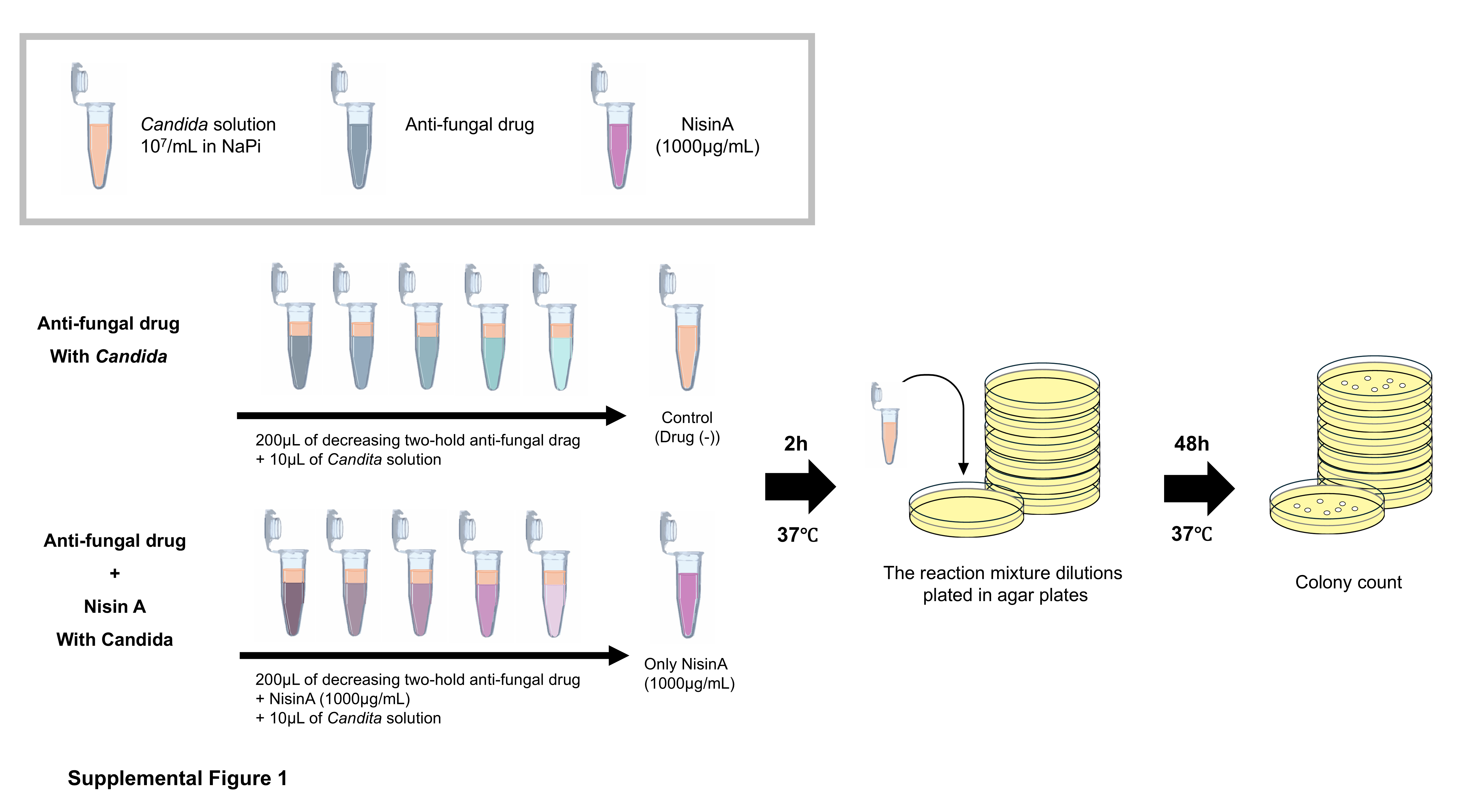

Supplement: Supplementary file 1 [file dentistry-13-00160-s001.zip › Supplemental figure S1.tif]

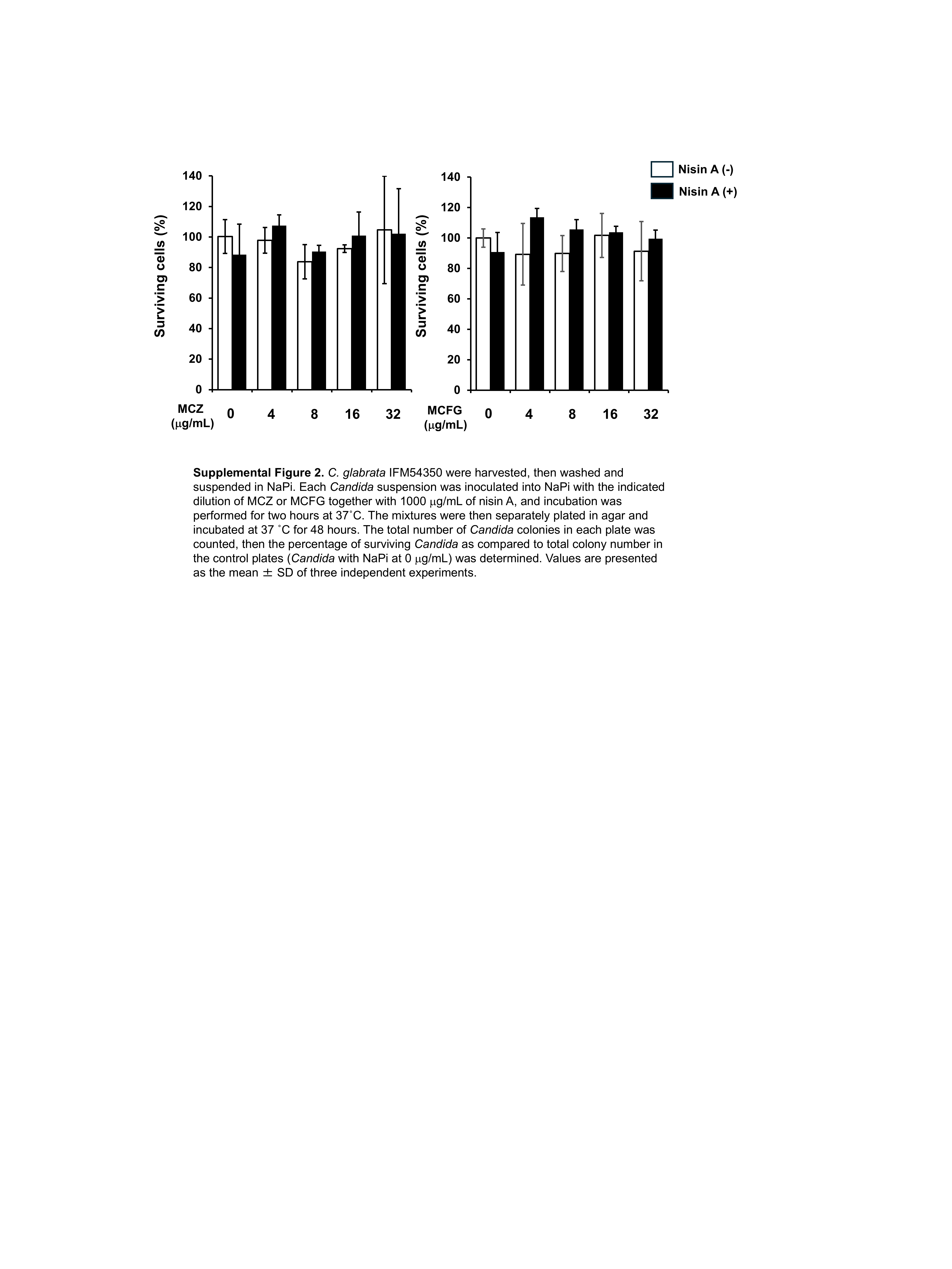

Supplement: Supplementary file 1 [file dentistry-13-00160-s001.zip › Supplemental Figure S2.TIF]
